# C/EBPβ: A transcription factor associated with the irreversible progression of Alzheimer's disease

**DOI:** 10.1111/cns.14721

**Published:** 2024-04-21

**Authors:** Qing Yao, Chubing Long, Pengcheng Yi, Guangyong Zhang, Wei Wan, Xiuqin Rao, Jun Ying, Weidong Liang, Fuzhou Hua

**Affiliations:** ^1^ Department of Anesthesiology The Second Affiliated Hospital of Nanchang University Nanchang City Jiangxi Province China; ^2^ Key Laboratory of Anesthesiology of Jiangxi Province Nanchang City Jiangxi Province China; ^3^ Department of Anesthesiology The First Affiliated Hospital of Gannan Medical University Ganzhou Jiangxi Province China

**Keywords:** Alzheimer's disease, C/EBPβ, neurodegenerative disease, therapy, transcription

## Abstract

**Background:**

Alzheimer's disease (AD) is a neurodegenerative disorder distinguished by a swift cognitive deterioration accompanied by distinctive pathological hallmarks such as extracellular Aβ (β‐amyloid) peptides, neuronal neurofibrillary tangles (NFTs), sustained neuroinflammation, and synaptic degeneration. The elevated frequency of AD cases and its proclivity to manifest at a younger age present a pressing challenge in the quest for novel therapeutic interventions. Numerous investigations have substantiated the involvement of C/EBPβ in the progression of AD pathology, thus indicating its potential as a therapeutic target for AD treatment.

**Aims:**

Several studies have demonstrated an elevation in the expression level of C/EBPβ among individuals afflicted with AD. Consequently, this review predominantly delves into the association between C/EBPβ expression and the pathological progression of Alzheimer's disease, elucidating its underlying molecular mechanism, and pointing out the possibility that C/EBPβ can be a new therapeutic target for AD.

**Methods:**

A systematic literature search was performed across multiple databases, including PubMed, Google Scholar, and so on, utilizing predetermined keywords and MeSH terms, without temporal constraints. The inclusion criteria encompassed diverse study designs, such as experimental, case–control, and cohort studies, restricted to publications in the English language, while conference abstracts and unpublished sources were excluded.

**Results:**

Overexpression of C/EBPβ exacerbates the pathological features of AD, primarily by promoting neuroinflammation and mediating the transcriptional regulation of key molecular pathways, including δ‐secretase, apolipoprotein E4 (APOE4), acidic leucine‐rich nuclear phosphoprotein‐32A (ANP32A), transient receptor potential channel 1 (TRPC1), and Forkhead BoxO (FOXO).

**Discussion:**

The correlation between overexpression of C/EBPβ and the pathological development of AD, along with its molecular mechanisms, is evident. Investigating the pathways through which C/EBPβ regulates the development of AD reveals numerous multiple vicious cycle pathways exacerbating the pathological progression of the disease. Furthermore, the exacerbation of pathological progression due to C/EBPβ overexpression and its molecular mechanism is not limited to AD but also extends to other neurodegenerative diseases such as amyotrophic lateral sclerosis (ALS), Parkinson's disease (PD), and multiple sclerosis (MS).

**Conclusion:**

The overexpression of C/EBPβ accelerates the irreversible progression of AD pathophysiology. Additionally, C/EBPβ plays a crucial role in mediating multiple pathways linked to AD pathology, some of which engender vicious cycles, leading to the establishment of feedback mechanisms. To sum up, targeting C/EBPβ could hold promise as a therapeutic strategy not only for AD but also for other degenerative diseases.

## Graphical abstract

C/EBPβ overexpression exacerbates the pathologic progression of AD, primarily by promoting neuroinflammation and mediating transcriptional regulation of several disease‐associated proteins, such as δ‐secretase and APOE4. Additionally, C/EBPβ stimulates the upregulation of ANP32A and TRPC1, leading to elevated levels of phosphorylated Tau, while inhibiting the expression of FOXO, which is associated with genes involved in longevity. The in‐depth research of the pathway through which C/EBPβ regulates the development of AD also reveals multiple vicious cycle pathways between C/EBPβ overexpression and the pathological progression of AD.

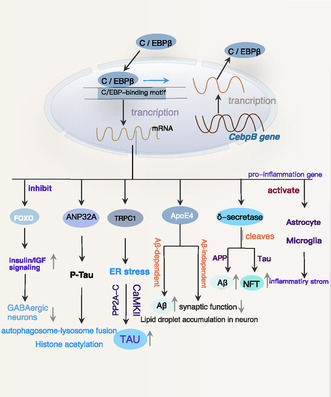

## 1 BACKGROUND

AD, a neurodegenerative condition marked by a gradual deterioration of cognitive abilities, stands as a prominent cause of mortality among individuals aged 65 and older. Its incidence escalates in tandem with the aging global population.^1^ AD is typified by distinctive pathological hallmarks, encompassing extracellular Aβ (β‐amyloid) peptides, neuronal neurofibrillary tangles (NFTs), persistent neuroinflammation, and synaptic degeneration.^2^ Aβ pathology arises from the aberrant sequential cleavage of amyloid precursor protein (APP) by β‐site APP cleaving enzyme 1 (BACE1) and γ‐secretase. This process leads to the accumulation of Aβ peptides, culminating in the formation of Aβ oligomers, fibrils, and plaques. NFTs primarily consist of hyperphosphorylated and truncated tau protein, which plays a crucial role in microtubule stabilization.^3^ A majority of patients manifest late‐onset Alzheimer's disease, a non‐hereditary, multifactorial condition lacking a singular genetic etiology. However, prevailing evidence underscores the significant role of APOE4 in disease pathogenesis.^4^ While age stands as the foremost risk factor for AD, several diseases and lifestyle factors elevate the susceptibility to this condition. These factors encompass traumatic brain injury, diabetes, hypertension, obesity, and various metabolic syndromes.^5^ Individuals with AD exhibit detectable elevation of inflammation in the cerebrospinal fluid (CSF), reflecting immune activation in the early stages of AD pathology.^6^ Research scholars have shown a significant interest in investigating the mechanisms underlying the onset and progression of AD. As our comprehension of AD has grown at the protein level, there has been a surge in studies investigating the generation of AD‐related pathologies through gene transcription processes in recent years.

The C/EBPs consist of a family comprising six transcription factor isotypes: C/EBPα, C/EBPβ, C/EBPδ, C/EBPε, C/EBPγ, and C/EBPζ.^7^ C/EBPs act as pleiotropic transcription activators of various genes involved in energy metabolism, cell differentiation, and inflammation, often acting in synergy with other transcription factors.^8^, ^9^ C/EBPβ is evolutionarily conserved among species and expressed in multiple organ systems.^10^, ^11^, ^12^ The gene is expressed as a single transcript that can be translated into three subtypes: the full‐length liver‐enriched activator protein (38‐kDa LAP1), the 34‐kDa liver‐enriched activator protein (LAP2), and the 21‐kDa liver‐enriched inhibitory protein (LIP).^13^ The upregulation of C/EBPβ‐activated isotype full/LAP usually precedes the upregulation of inhibitory isoform LIP.^14^ C/EBPβ mRNA is expressed in different regions of the adult rodent brain.^15^ C/EBPβ promoted neurite regeneration and growth after nerve injury,^15^, ^16^ and promoted the expression of Aβ scavenger CD36 at the transcriptional level.^17^ C/EBPβ has been shown to play a role in synaptic plasticity and memory formation, mainly in the hippocampus, neuronal differentiation, and hippocampal neurogenesis.^18^

Recent research has demonstrated that there is an overexpression of C/EBPβ in patients with a variety of neurodegenerative diseases, and it is closely associated with the irreversible progression of AD. Acting as an enhancer‐binding protein, C/EBPβ facilitates gene expression by selectively binding to specific gene sequences. Genes linked to AD, including pro‐inflammatory, *LMGN*, and *APOE* genes, contain distinct sequences susceptible to C/EBPβ binding.^19^, ^20^, ^21^ Furthermore, C/EBPβ mediates the transcription and expression of calcium‐related channel proteins, inducing endoplasmic retinal stress. This, in turn, leads to the dysregulation of phosphatase and protease activities,^22^ FOXO inhibition, GABA neuron degeneration, and inactivation,^23^ and even influences histone acetylation,^24^ ultimately expediting AD progression and perpetuating an irreversible deleterious loop. C/EBPβ could emerge as a pivotal site and a promising therapeutic target in the pathogenesis of AD.

## 2 THE MOLECULAR PATHWAY MECHANISMS OF C/EBPβ REGULATING THE IRREVERSIBLE DEVELOPMENT OF AD

C/EBPβ exhibits elevated expression levels within the hippocampus, cortex, and cerebellum, correlating significantly with neuronal developmental processes. During central nervous system (CNS) development, in addition to playing multiple roles in diverting cortical precursors to neuronal fate and benefiting neurogenesis, the expression of C/EBPβ in the adult hippocampus has been shown to be primarily localized in the nucleus of granular neurons of the dentate gyrus and to play a key role in regulating the proliferation and differentiation of these cells in vitro and in vivo.^25^, ^26^, ^27^ The comprehensive investigation of C/EBPβ's cellular and subcellular localization in the healthy CNS is yet to be undertaken. Notably, C/EBPβ has been directly associated with the progression of AD in humans. Protein levels are heightened in the brains of AD patients in comparison to those of healthy controls. Furthermore, the transcript of *Cebpb* (which encodes C/EBPβ) exhibits greater abundance in the brains of aged AD mouse models than in those of aged control mice.^19^ The levels of C/EBPβ increase in neurons in an age‐dependent fashion.^28^ Many pro‐inflammatory genes contain the underlying C/EBPβ consensus sequence, which plays a crucial role in central nervous inflammation. In AD, glial cells manifest heightened C/EBPβ levels due to sustained upregulation of pro‐inflammatory cytokines in the cortex, with a particularly notable increase observed in microglia.^29^, ^30^ Microglia exhibit heightened expression and nuclear localization of C/EBPβ. Furthermore, immunohistochemical studies have validated robust colocalization between C/EBPβ‐immunoreactive microglia and Aβ deposits and Aβ immunoreactive microvessels, along with pronounced immunoreactivity in pathologically vulnerable regions of the AD brain, contrasting with comparatively faint staining in corresponding areas of the non‐demented (ND) brain or pathologically spared regions of the AD brain (e.g., cerebellum).^31^ Numerous studies have substantiated the pivotal role of C/EBPβ in the pathogenesis of AD, hastening its irreversible progression via diverse pathways (Table 1).

**TABLE 1:** The molecular pathways of Alzheimer's disease progression are regulated by C/EBPβ.

Molecular pathways	Mechanisms	Results
C/EBPβ‐inflammatory factors	Inflammatory factors activate glial cells, leading to an upregulation of C/EBPβ expression and nuclear translocation. C/EBPβ subsequently binds to the promoters of inflammatory factor genes, thereby promoting the expression of additional inflammatory factors	inflammatory factors↑ Neuroinflammation↑
C/EBPβ‐δ‐secretase	C/EBPβ binds to the promoter of the *LMGN* gene, thereby enhancing the expression of δ‐secretase, an enzyme responsible for cleaving both APP and Tau	Aβ peptides↑ NFTs↑
C/EBPβ‐APOE4	C/EBPβ acts as a specific transcription factor for APOE to promote its gene transcription. ApoE4 exacerbates AD progression via both Aβ‐independent and Aβ‐dependent pathways	Aβ peptides↑ synaptic function↓ Lipid droplet accumulation in neuron↑ C/EBPβ↑
C/EBPβ‐TRPC1‐SOCE	C/EBPβ binds to the promoter sequence of TRPC1, thereby promoting the transcription of TRPC1, subsequently elevating the steady‐state calcium ion concentration in neurons ([Ca^2+^] I), augmenting ER stress, disrupting the balance of protein kinases and phosphatases, and worsening tauopathy	hTau↑ p‐Tau↑
C/EBPβ‐ANP32A	The activation of C/EBPβ prompts the transcription of the ANP32A gene, inhibits histone acetylation, and promotes the phosphorylation of Tau	p‐Tau↑ autophagosome‐lysosome fusion↓ gene expression of long‐term memory↓
C/EBPβ‐FOXO1	C/EBPβ suppresses the promoter activity of REST and FOXO1, diminishes their transcription, promotes Aβ aggregation toxicity, and triggers the death of GABAergic neurons	GABA neuron↓ Aβ↑ AEP↑

### 2.1 The role of C/EBPβ in neuroinflammation and its functional interaction with inflammatory mediators

Neuroinflammation plays a pivotal role in the propagation of several neurodegenerative disorders and stands as a significant contributing factor in the pathogenesis and advancement of AD. In the context of CNS inflammation, we primarily refer to the response of microglia and astrocytes to disruptions in homeostasis, which entail profound alterations in gene expression. These alterations frequently stem from cellular recognition of Aβ or other damage‐ or pathogen‐associated molecular patterns (collectively known as DAMPs or PAMPs).^32^, ^33^ Neuroglial cells assume a crucial role in various neurobiological processes, including neurogenesis, the preservation of neuronal well‐being, and the formation of neuronal circuits. They accomplish these functions through a spectrum of activities, including anti‐inflammatory responses, phagocytosis, steroid secretion, free radical scavenging, and cellular repair mechanisms.^34^, ^35^ Neuroinflammation manifests in a state of heightened activation among glial cells, characterized by the production of pro‐inflammatory cytokines. The excessive activation of inflammatory molecules intensifies neuroinflammation within the brain, precipitating the onset of AD and resulting in synaptic dysfunction, neuronal demise, and the impediment of neurogenesis, ultimately leading to irreversible cerebral damage.^36^ Neuroinflammation in the context of neurodegenerative diseases frequently manifests as a persistent and unresolved chronic process, contributing significantly to disease progression.^37^ Presently, a multitude of studies have underscored the involvement of C/EBPβ in the regulation of neuroinflammation, emphasizing its central role in the promotion of inflammatory gene expression. Notably, numerous pro‐inflammatory gene promoters harbor putative C/EBPβ consensus sequences.^38^ Moreover, the expression and activation of C/EBPβ are subject to regulation by diverse extracellular signals, frequently involving intricate modulation by proinflammatory cues like IL‐6, IL‐43, and LPS.^39^

#### 2.1.1 C/EBPβ and astrocyte activation

**FIGURE 1:**
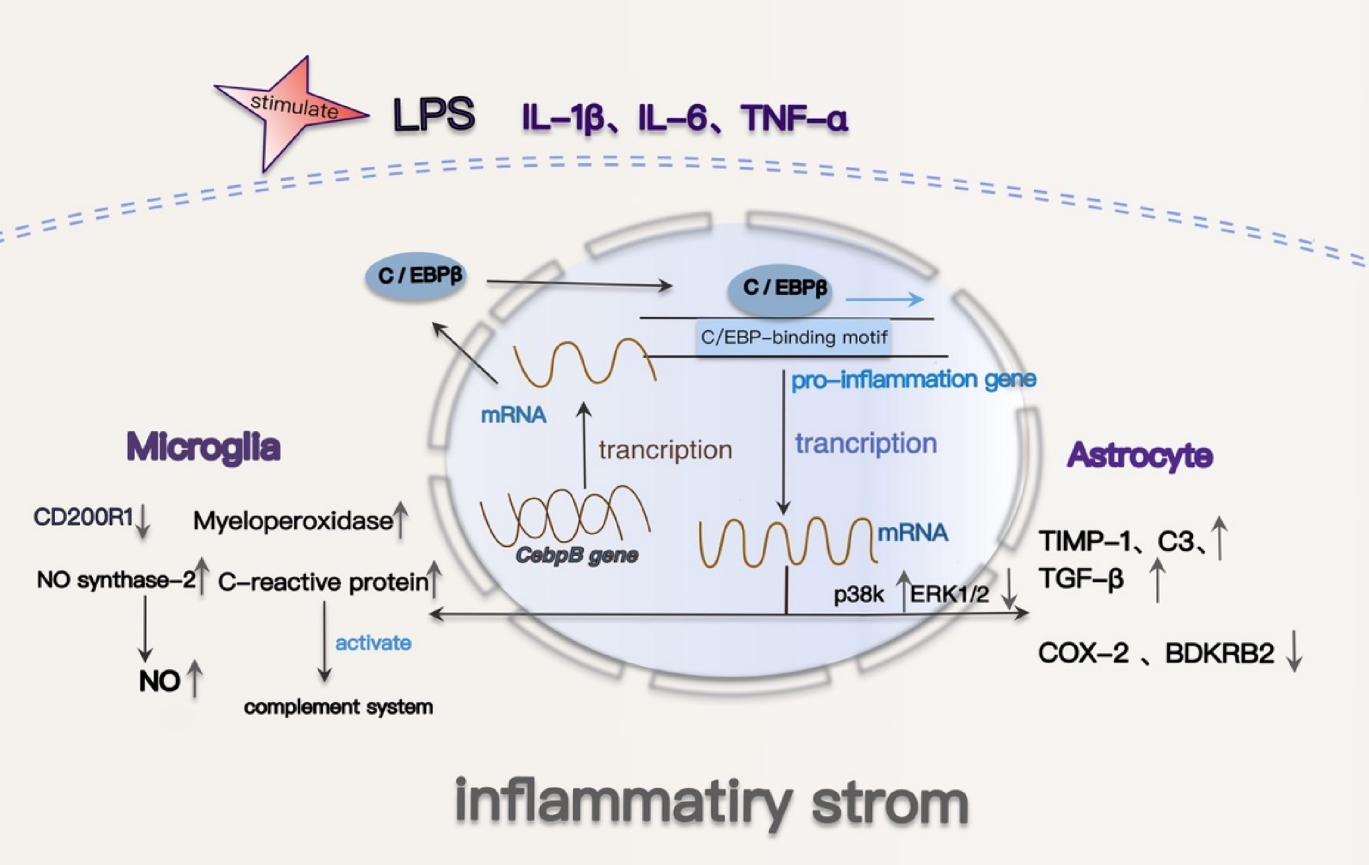
C/EBPβ regulates signaling pathways associated with brain inflammation in AD. C/EBPβ and astrocyte activation: After the activation of astrocytes, the expression of C/EBPβ increases and migrates to the nucleus to regulate gene expression in the nucleus as a transcription factor. C/EBPβ inhibited some ERK1/2 regulatory genes, activated P38K regulatory genes, downregulated ERK1/2 expression, and increased p38K expression, respectively, BDKRB2 expression retention and cox‐2 expression increase in astrocytes. C/EBPβ can also bind to C3, TIMP‐1, and TGF‐β1 promoters to promote their expression. C/EBPβ and microglia activation: After treatment with LPS, IL‐1β, IL‐6, or TNF‐α, C/EBPβ in microglia cells was significantly increased and its transfer to the nucleus was increased. C/EBPβ was bound to pro‐inflammatory gene promoters in a stimulatory and gene‐dependent manner to promote the expression of inflammatory genes in glia activation. iNOS, myeloperoxidase, C‐reactive protein, M‐CSF, and other promoters showed C/EBPβ‐binding sites. Inflammatory factors activate glial cells, induce the increase of C/EBPβ expression, and promote the increase of the expression of more inflammatory factors.

The activation of astrocytes is influenced by various stimuli, including lipopolysaccharide (LPS), interleukin‐1β (IL‐1β), and tumor necrosis factor‐alpha (TNF‐α), which induce the expression of C/EBPβ and C/EBPδ genes in primary astrocytes, thereby promoting astrocyte activation.^40^, ^41^ Notably, LPS concentration dependently upregulates the expression of IL‐1β and TNF‐α in astrocytes.^42^ The pro‐inflammatory cytokine interleukin‐1β (IL‐1β) is one of the most potent and characteristic signals triggering astrocyte activation in most neurodegenerative diseases, mediating neuroinflammation through activation of glial cells and subsequent changes in gene expression.^43^ After IL‐1β‐mediated activation of glial cells, astrocyte C/EBPβ expression is induced by IL‐1β and localized to the nucleus, where it acts as a transcription factor to regulate gene expression in the nucleus.^40^, ^44^ Specifically, IL‐1β alters the mRNA expression of 32% (29/92) of inflammatory genes mediated by IL‐1β, with C/EBPβ regulating the expression of 52% (17/29) of these genes. In IL‐1β‐activated astrocytes, expression levels of nos‐2 and intercellular adhesion molecule‐1 (ICAM‐1) are elevated and decreased, respectively, compared to C/EBPβ‐deficient astrocytes.^45^, ^46^ Additionally, C/EBPβ inversely regulates the expression of cyclooxygenase‐2 (COX‐2) and bradykinin receptor B2 (BDKRB2).^47^ Specifically, increased C/EBPβ expression enhances COX‐2 expression while diminishing BDKRB2 expression. IL‐1β signaling increases *BDKRB2* and *COX‐2* mRNA in astrocytes via extracellular regulated kinase (ERK) 1/2 and p38 kinase (p38K), respectively.^48^ P38K inhibition blocked IL‐1β‐mediated C/EBPβ expression in astrocytes, whereas ERK1/2 inhibition enhanced expression.^49^ This suggests that C/EBPβ may inhibit some ERK1/2‐regulated genes while activating p38K‐regulated genes, thereby influencing gene regulation.^48^ Overexpression of C/EBPβ isoforms LAP1 and LAP2 also have profound effects on the induction of C3‐related gene promoter activity.^50^, ^51^ In response to nerve injury, astrocytes secrete tissue inhibitors of metalloproteinase‐1 (TIMP‐1). Altered TIMP‐1 levels have implications in various CNS diseases, including AD. Interestingly, overexpression of C/EBPβ in primary human astrocytes substantially enhances the activity of the −1718/+988 region of the TIMP‐1 promoter, leading to an increased TIMP‐1 expression.^44^ Furthermore, C/EBPβ increases transforming growth factor‐β1 (TGF‐β1) promoter activity by binding to C/EBPβ binding sites in the −160 to −100 region of the TGF‐β1 promoter. This induction triggers the TGF‐β1/Smads signaling pathway, subsequently promoting astrocyte proliferation, which perpetuates the organism's state of disease.^52^ In summary, C/EBPβ, through its functional interactions with inflammatory factors, exerts regulatory control over a multitude of human astrocyte‐related genes during neuroinflammation (Figure 1).

#### 2.1.2 C/EBPβ and microglia activation

Elevated levels of C/EBPβ in activated microglia govern the expression of pivotal pro‐inflammatory genes, exerting a significant role in gene regulation within activated microglia.^53^ Furthermore, the percentage of C/EBPβ‐immunopositive cells linked to Aβ peptides markedly exceeded those unrelated to Aβ plaques. Notably, strong co‐localization of C/EBPβ immunoreactivity occurred with Aβ deposits and Aβ‐immunoreactive microvessels, with concurrent co‐localization of C/EBPβ and microglia in the presence of Aβ peptides.^31^ Microglial C/EBPβ also responds to the upregulation of C/EBP‐inducing cytokines or lipopolysaccharides, leading to upregulation and nuclear localization. Following treatment with C/EBP‐inducing pro‐inflammatory cytokines IL‐1β, IL‐6, or TNF‐α, C/EBPβ levels in microglia significantly increased, accompanied by enhanced nuclear translocation, akin to the effect of LPS treatment.^31^ Treatment with lipopolysaccharide (LPS) or the combination of LPS and interferon γ (IFNγ) induced significant elevations in C/EBPβ mRNA and protein levels, along with enhanced DNA binding in glial cultures. C/EBPβ assumes a pivotal role as a component of the C/EBPs DNA‐binding complex during LPS and LPS+ IFNγ‐induced glial activation. During glial activation, C/EBPβ selectively binds to pro‐inflammatory gene promoters in a stimulatory and gene‐dependent fashion, thereby facilitating the expression of inflammatory genes.^54^ Pro‐inflammatory factors promote the upregulation and translocation of C/EBPβ to the nucleus, subsequently fostering the production of inflammatory factors. Extensive evidence has demonstrated the neurocytotoxicity of the inducible nitric oxide synthase (iNOS) mechanism in the AD brain, with NO production assuming a prominent role in the neurotoxicity attributed to activated microglia.^55^ Upregulation of C/EBPβ results in heightened iNOS expression via direct transcriptional mechanisms.^56^, ^57^ The absence of C/EBPβ in microglia led to diminished expression of IL‐1β and NO synthase‐2, resulting in reduced NO production. Additionally, it entirely abolished the neurotoxicity induced by co‐cultured microglia treated with LPS + IFNγ when in contact with neurons.^54^ Moreover, C/EBPβ appears to downregulate CD200R1 receptor expression in microglia during inflammation, rendering them less responsive to inhibitory CD200 ligands on neurons.^58^ The transcriptional regulation of macrophage colony‐stimulating factor (M‐CSF), a growth factor linked to inflammation and Aβ peptide formation in microglia, is orchestrated by C/EBPβ.^59^, ^60^ C‐reactive protein possesses the capability to activate the complement system and harbors a binding site for C/EBPβ within its promoter.^61^ Myeloperoxidase similarly contains a promoter site for C/EBPβ.^62^ The degradation of C/EBPβ in microglia resulted in the inhibition of neuroinflammation.^19^ C/EBPβ plays an essential role in upregulating inflammatory gene expression and inducing neurotoxicity in microglia during neurodegenerative diseases (Figure 1). It serves as a pivotal transcription factor for reprogramming microglial activation, making it a potential therapeutic target to mitigate neuronal injury caused by neuroinflammation.

### 2.2 The C/EBPβ‐δ‐secretase axis is upregulated, resulting in increased levels of Aβ and NFT in AD

**FIGURE 2:**
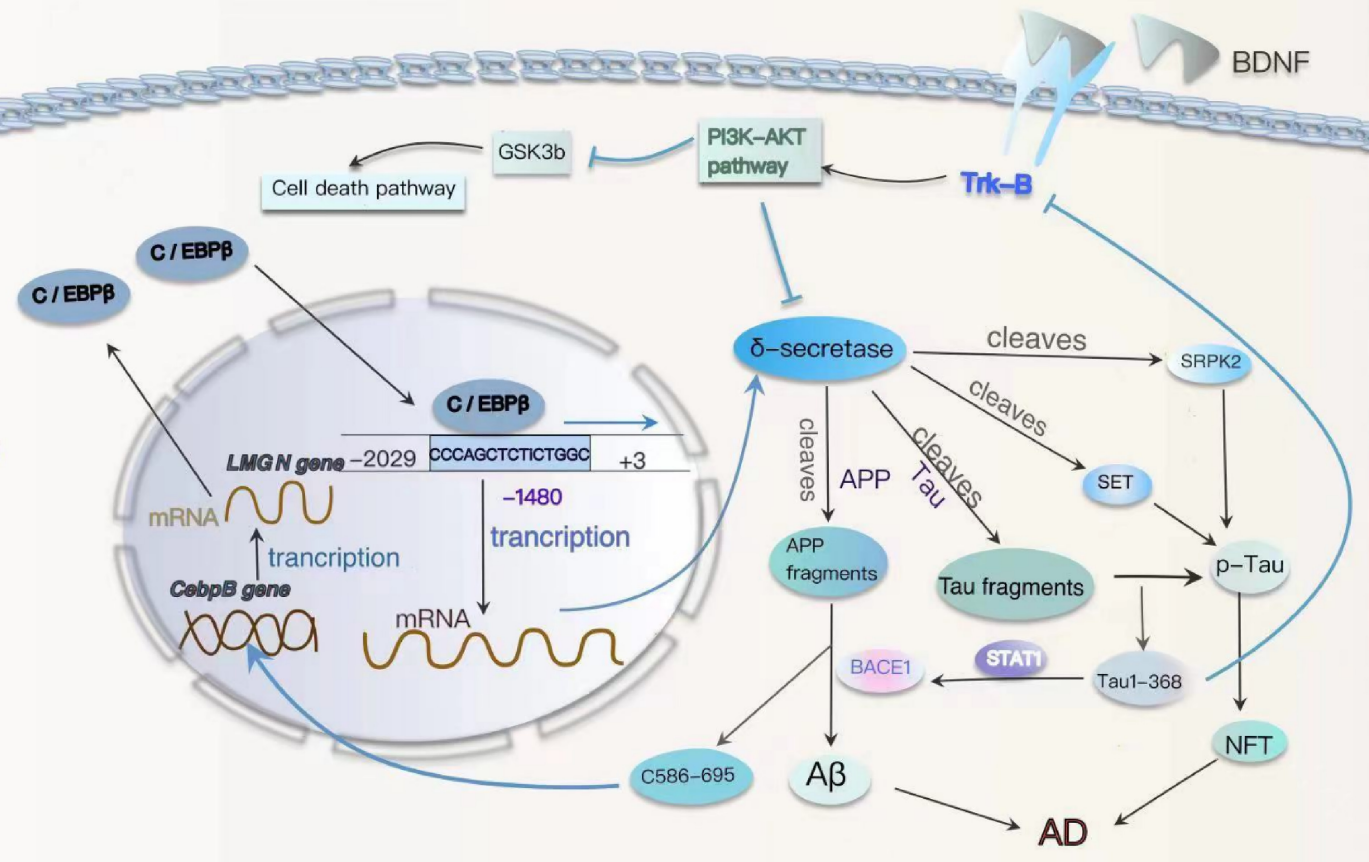
The molecular mechanisms underlying the C/EBPβ‐δ‐secretase signaling pathway expedite the progression of AD. The expression of δ‐secretase is regulated by the transcription factor C/EBPβ, which is regulated by the BDNF/TrkB signaling pathway. δ‐secretase is phosphorylated by Akt, which inhibits its activity, and SRPK2, which enhances its activity. The δ‐secretase cleaves APP and promotes the production of Aβ. The δ‐secretase also cleaves Tau, producing fragments that are prone to aggregation. The tau fragment derived from δ‐secretase‐enhanced BACE1 activity, which further promoted the production of Aβ. δ‐secretase‐derived SET fragments inhibit tau dephosphorylation, while δ secretase‐derived SRPK2 fragments affect tau selective splicing. Aβ and APP C586‐695 (C110) fragments ultimately stimulate the transcription of genes associated with AD by binding to and activating C/EBPβ. All of these pathways promote tau aggregation and the occurrence of AD.

Asparagine endopeptidase (AEP), a cysteine protease, has been newly identified as δ‐secretase. It cleaves APP and Tau in an age‐dependent manner, leading to the formation of amyloid plaques and NFTs in AD.^63^ δ‐secretase cleaves APP at residues N373 and N585 on the extracellular structural domain, enhancing Aβ production by BACE1.^64^ It also cleaves Tau at sites N255 and N368, leading to accelerated Tau hyperphosphorylation and the subsequent accumulation of NFT.^65^ Importantly, the tau (1–368) fragment is neurotoxic^66^ and binds to, activating the transcription factor STAT1, thereby further up‐regulating BACE1 transcription and Aβ production.^67^ Besides APP and tau, δ‐secretase also cleaves polyanionic serine‐arginine protein kinase 2 (SRPK2), a protein crucial in RNA splicing through the phosphorylation of SR splicing factors.^68^ During tau mRNA splicing, SRPK undergoes δ‐secretase cleavage, nuclear translocation, and an increase in kinase activity. This results in enhanced exon 10 attachment and an imbalance in 4R‐tau and 3R‐tau expression, ultimately promoting tau aggregation in tauopathy.^69^ Additionally, δ‐secretase cleaves SET, which functions as a DNase inhibitor, PP2A inhibitor, and a regulator of tau phosphorylation.^70^ Fragments derived from δ‐secretase cleavage of SET lose their DNase inhibitor activity, induce genomic DNA incision, leading to neuronal cell death,^71^ and inhibit PP2A activation, which triggers hyperphosphorylation and tau aggregation in AD^72^ (Figure 2). Overexpression of δ‐secretase‐derived SET fragments in the brain similarly reproduces the key features of AD in rats.^73^ In conclusion, it is widely validated that δ‐secretase plays an important role in the pathogenesis of AD.

Further investigation into the regulatory mechanism of δ‐secretase has demonstrated that complete depletion of C/EBPβ results in the total elimination of δ‐secretase expression. Within the −2029 to +3 base pair region of the *LMGN* (δ‐secretase gene) promoter, the highest transcriptional activity is observed. C/EBPβ substantially enhances *LMGN* promoter activity and associates with the GAATTCAGAGGC sequence located 1480 bases upstream of the *LMGN* promoter, facilitating *LMGN* mRNA transcription^74^ (Figure 2). C/EBPβ serves as a crucial transcription factor that mediates the expression of δ‐secretase, thereby promoting the accelerated development of Alzheimer's disease. The levels of δ‐secretase protein in both the brain and spinal cord exhibit age‐dependent increases.^65^ In the CNS, C/EBPβ modulates the expression of δ‐secretase in a time‐dependent fashion.^75^, ^76^ Simultaneously, both Aβ and the δ‐secretase‐truncated APP C586‐695 (C110) fragments stimulate the transcription of Alzheimer's disease‐related genes by binding to and activating C/EBPβ, which encompasses δ‐secretase.^77^, ^78^ C/EBPβ plays a pivotal role in regulating the transcription and activation of *LMGN* mRNA during aging, thereby mediating the initiation and progression of AD pathology.^79^ In parallel, δ‐secretase engages in a feedback loop by cleaving APP and generating a protein‐hydrolyzed APP C586‐695 fragment, which selectively binds to and enhances the transcriptional activity of C/EBPβ.^77^ C/EBPβ serves as the biological link in the detrimental cycle between δ‐secretase and Aβ in the context of AD.

Through the C/EBPβ‐δ‐secretase axis, C/EBPβ binds to enhancers in *LMGN* gene transcription and acts as a transcription factor, leading to an elevation in δ‐secretase expression, thus expediting the progression of AD. Furthermore, the C/EBPβ‐δ‐secretase axis mediates the interaction between numerous exogenous and endogenous risk factors and the development of AD. Intestinal inflammation or microbiota alterations activate the C/EBPβ‐δ‐secretase axis, initiating AD pathology in the gut, which subsequently ascends to the brain via the vagus nerve.^28^, ^80^, ^81^ The concept of the gut‐brain axis has garnered increasing attention, and the C/EBPβ‐δ‐secretase axis further strengthens the connection between intestinal inflammation or microbiota and neurodegenerative diseases. Atherosclerosis (ATH) and AD are both age‐dependent inflammatory diseases associated with infiltrating macrophages, vascular pathology, and shared molecular factors. In recent experiments, it has been shown that C/EBPβ‐AEP signaling links atherosclerosis (ATH) to AD by mediating vascular pathology.^82^ Inflammatory responses triggered by a high‐fat diet (HFD) activate neuronal C/EBPβ‐AEP signaling, resulting in the development of AD pathology and cognitive impairments.^83^ Traumatic brain injury (TBI) induces the activation of the transcription factor C/EBPβ, leading to an upregulation of δ‐secretase expression. This, in turn, contributes to the pathogenesis of AD by fostering the production of Aβ, Tau hyperphosphorylation, and the induction of neuroinflammation and neurotoxicity.^84^ The expression level of C/EBPβ exhibited a negative correlation with BDNF/pTrkB signaling. Decreased BDNF/TrkB signaling results in elevated inflammatory cytokines and the activation of the JAK2/STAT3 pathway, ultimately leading to an upregulation of C/EBPβ and δ‐secretase, consequently enhancing proteolytic APP and Tau fragmentation.^66^, ^85^ Over recent years, numerous studies have substantiated C/EBPβ and δ‐secretases as promising therapeutic targets for AD.

### 2.3 C/EBPβ mediates the transcription of APOE4, the key gene in AD

**FIGURE 3:**
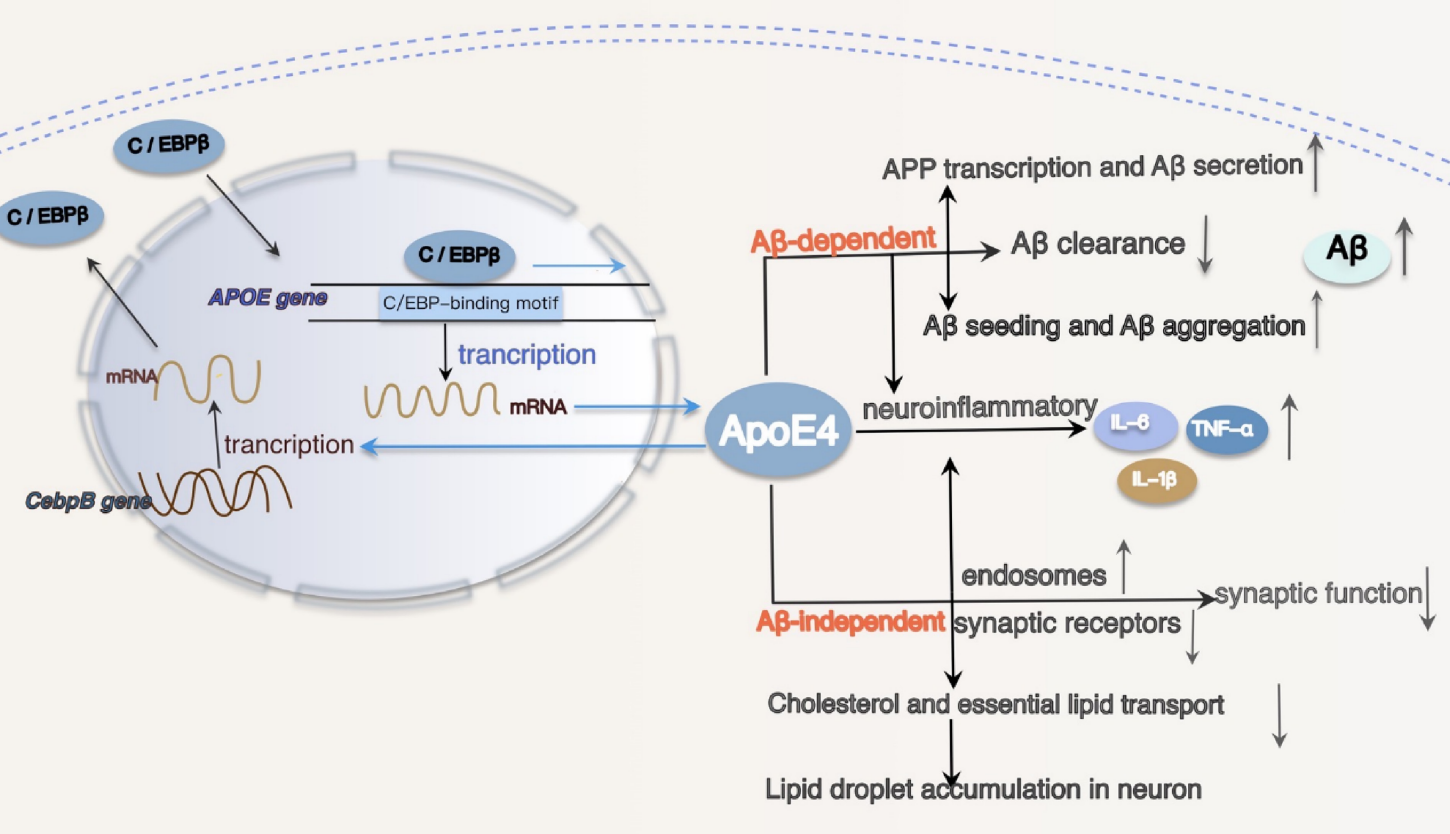
C/EBPβ mediates the expression of APOE4, leading to the accumulation of lipid droplets and Aβ. The transcription factor C/EBPβ binds and interacts with the C/EBP‐binding motifs within the APOE promoter −305 to +93 fragment, acting as a specific transcription factor for APOE and promoting its gene transcription. ApoE4 can influence lipid transport through an Aβ‐independent pathway, leading to mitochondrial damage and increased reactive oxygen species. Additionally, it can enhance Aβ production through an Aβ‐dependent pathway, concurrently amplifying neuroinflammatory responses. Conversely, ApoE4 strongly activates C/EBPβ, subsequently enhancing δ‐secretase cleavage of the increased APP.

ApoE, a glycoprotein primarily expressed in astrocytes, microglia, neurons, and the choroid plexus, functions as a lipid transporter, particularly for cholesterol and cholesteryl esters.^86^ In humans, the *APOE* gene exists in three polymorphic alleles (ε2, ε3, and ε4). *APOE* isoforms have been found to influence Aβ and Tau deposition in the brain, with *APOE4* being the most potent genetic risk factor for AD.^87^ It substantially elevates the risk of both early‐onset and late‐onset AD in a dose‐dependent manner.^88^ ApoE4‐related pathogenesis can be categorized into Aβ‐related and Aβ‐independent mechanisms (Figure 3). ApoE plays a pivotal role in lipid transport and cholesterol homeostasis within the brain. Compared with other ApoE isoforms, ApoE4 isoforms show a low lipid state, are prone to form non‐lipid‐containing ApoE4 aggregates, and have poor lipid turnover between neurons and astrocytes, which leads to the accumulation of neuronal lipid droplets.^89^ This promotes mitochondrial damage and reactive oxygen species (ROS) production.^90^ In addition, ApoE4 also promotes the formation of endosomes and the degradation of synaptic receptors, including AMPA and NMDA, resulting in impaired synaptic function.^89^, ^91^ Peripheral apoE4 may also have potentially negative effects on the pathological processes in brain development.^92^ ApoE can influence the neuroinflammatory process through various mechanisms; however, *APOE4* astrocytes and microglia exhibit dysfunction in their response to inflammatory injury.^93^ Experimental results demonstrate that mice expressing the human ApoE4 isoform exhibit a higher presence of Aβ deposits and neuritic amyloid plaques compared to mice expressing other human ApoE isoforms.^94^ It has been reported that ApoE‐isoforms have an Aβ‐dependent effect on APP transcription and Aβ secretion. ApoE4 induces the strongest increase in APP transcription,^95^ and ApoE4 also through mitogen‐activated protein kinase signaling^96^ and Aβ seeding and Aβ aggregation into oligomers and fibrils, increasing Aβ production and aggregation,^97^ while apoE4 reduces Aβ clearance.^88^ Furthermore, ApoE4 exacerbates tau‐mediated pathogenesis, neurotoxic neuroinflammation, and neurodegeneration.^98^ For instance, it increases tau neurotoxicity through its binding to the monoamine transporter 2 (VMAT2), inhibits the active transport of neurotransmitters into synaptic vesicles, and ultimately results in degeneration of the Locus Coeruleus.^99^ In a word, the ApoE4 is a significant factor in the development of Alzheimer's disease occurred.

ApoE production can be stimulated by a range of transcription factors, regulatory elements, hormones, cytokines, and lipids.^100^ A positive correlation exists between C/EBPβ expression levels and ApoE levels, indicating a close involvement of C/EBPβ in regulating *APOE* transcription and protein expression.^101^ The *APOE* promoter harbors multiple putative C/EBPβ binding motifs. Notably, the −305 to +93 fragment, which contains just one C/EBP‐binding motif, exhibits transcriptional activity akin to that of the full‐length promoter. This fragment encompasses the primary C/EBPβ‐binding site on the *APOE* promoter.^20^ In the mouse model of AD, C/EBPβ governs ApoE expression across multiple cell types, encompassing neurons, astrocytes, and microglia. Additionally, it modulates Aβ‐induced ApoE transcription and protein expression, with a particular impact on ApoE4.^20^ C/EBPβ functions as an *APOE*‐specific transcription factor by binding and interacting with the C/EBP‐binding motif within the −305 to +93 fragment of the *APOE* promoter, facilitating the transcription of *APOE* genes (Figure 3).

Neurons express ApoE, especially in stress response, particularly in the context of AD.^102^, ^103^ C/EBPβ is primarily responsible for the Aβ‐induced upregulation of ApoE expression, and Aβ42 oligomers produce C/EBPβ upregulation in a dose‐dependent manner, resulting in increased ApoE and AEP levels.^20^ Remarkably, neuronal ApoE4 robustly triggers C/EBPβ activation, which, in turn, enhances δ‐secretase activity, leading to increased cleavage of APP and Tau in mice. This cascade promotes AD‐like pathology, with Thy1‐ApoE4/C/EBPβ mice exhibiting age‐dependent development of amyloid deposits, Tau aggregates, neurodegeneration, synaptic dysfunction, and cognitive impairment.^104^ Conversely, ApoE4 exacerbates neuroinflammation and elevates Aβ levels, both of which can further stimulate C/EBPβ activation.^40^, ^78^ ApoE4, in conjunction with 27‐hydroxycholesterol (27‐OHC), collaboratively activates C/EBPβ/δ secretase signaling in neurons, mediating AD pathogenesis, which is dependent on the neuronal secretion of Aβ and inflammatory cytokines.^105^ A feedback loop operates between C/EBPβ and ApoE4, wherein ApoE4 strongly activates C/EBPβ, contributing to pathological changes in AD. Conversely, overexpression of C/EBPβ preferentially mediates the effects of ApoE4. In conclusion, C/EBPβ serves as the principal transcription factor for *APOE*, notably *APOE4*, which represents a prominent genetic factor in AD and significantly contributes to the disease's development. C/EBPβ and ApoE reciprocally modulate and enhance their respective biological roles in the pathogenesis of AD. Breaking this detrimental cycle holds promise as an appealing strategy for intervening in the initiation and progression of AD.

### 2.4 Upregulation of C/EBPβ‐TRPC1‐SOCE signaling causes ER stress and dysregulation of protein enzymes and phosphatases, resulting in increased p‐Tau

Store‐operated calcium entry (SOCE) is of paramount importance in the CNS, and its dysregulation gives rise to neuroinflammation, synaptic dysfunction, and neuronal demise.^106^, ^107^, ^108^ SOCE constitutes a ubiquitous Ca^2+^ entry pathway, activated following the depletion of endoplasmic reticulum (ER) calcium stores, initiated by the stimulation of plasma membrane receptors linked to phosphatidylinositol 4,5‐bisphosphate (PIP2) hydrolysis and inositol trisphosphate (IP3) production.^109^ Key molecular constituents of the SOCE machinery encompass stromal interaction molecules (STIM1/2), Orai channels (ORAI2/3), and transient receptor potential channels (TRPC1‐7). Upon depletion of ER calcium stores, STIM1/2 detects reduced ER calcium levels, leading to oligomerization and subsequent translocation from ER‐like sites to the plasma membrane, where they interact with calcium‐conductive channels (ORAI and TRPC) to elicit calcium influx and replenish calcium stores.^110^, ^111^ TRPC1, the first identified mammalian TRPC protein, exhibits widespread expression in both neuronal and nonneuronal tissues and is currently the principal candidate component of the SOCE system, contributing significantly to SOCE in various cell types.^112^ Cumulative evidence underscores the indispensable role of the TRPC1‐SOCE pathway in the initiation and progression of neurodegenerative disorders.

**FIGURE 4:**
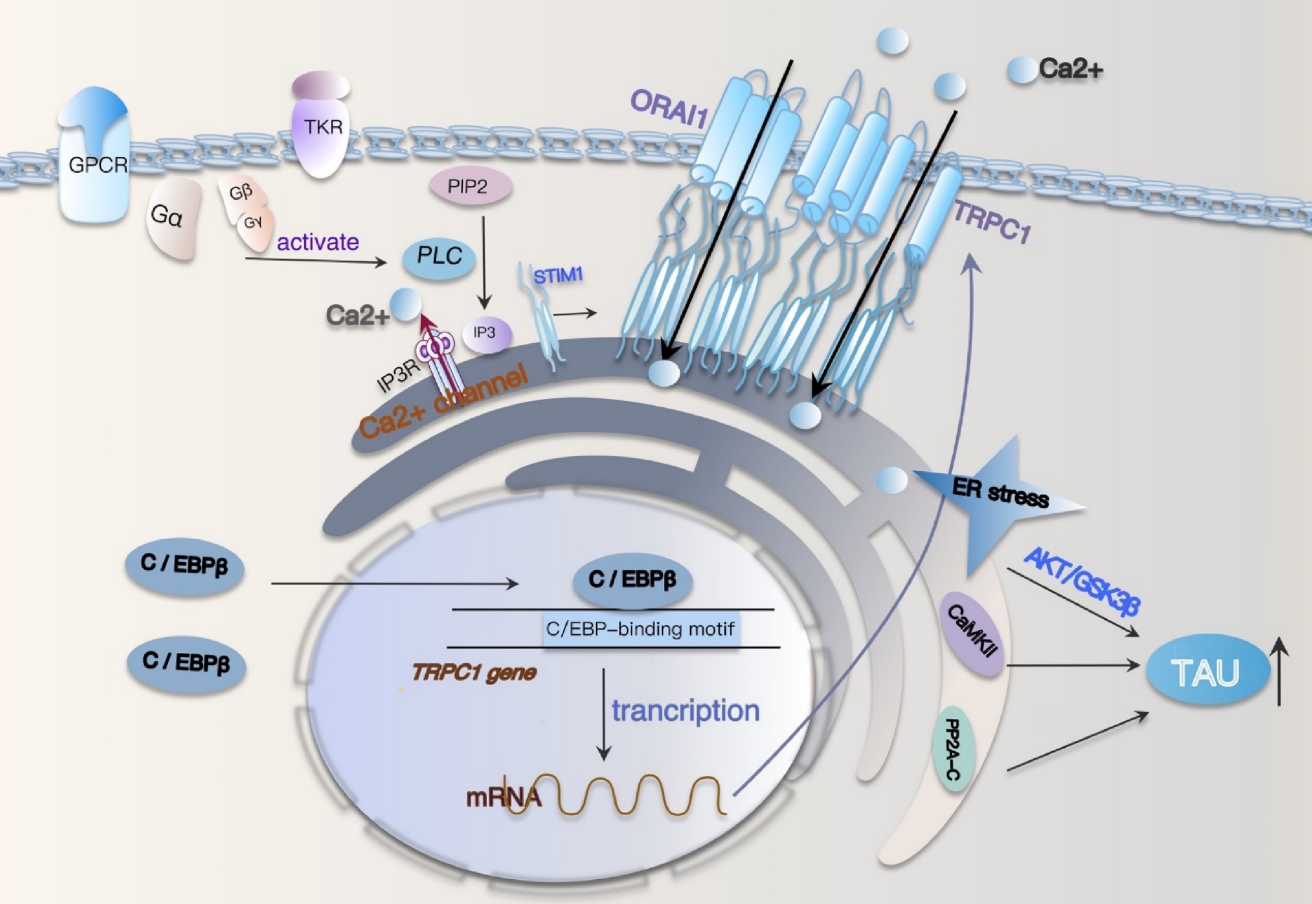
C/EBPβ‐TRPC1‐SOCE signaling causes ER stress and dysregulation of protein enzymes and phosphatases. After G protein‐coupled receptors and tyrosine receptors were activated, PLC was subsequently activated to generate IP3 from PIP2. As a calcium channel, IP3R binds to IP3 and is activated, resulting in calcium ion outflow. When Ca^2+^ storage in the ER is depleted, STIM1/2 senses a reduction in Ca^2+^ in the ER, which leads to oligomerization and subsequent transfer from ER‐like sites to the plasma membrane, where it interacts with calcium conduction channels (ORAIs and TRPCs) to induce Ca^2+^ influx and storage regeneration. In the context of hTau overexpression, activated C/EBPβ binds to the TRPC1 promoter sequence, promoting TRPC1 transcription, then increases neuronal steady‐state [Ca^2+^] I, enhances endoplasmic reticulum (ER) stress, imbalances protein kinases, and phosphatases, and exacerbates tauopathy. The C/EBPβ‐Trpc1‐soce‐signaling pathway in turn increases hTau, thus accelerating the progression of AD.

Overexpression of full‐length wild‐type human tau (known as hTau) is an early tau pathological hallmark observed in sporadic AD. The overexpression of intracellular tau can trigger TRPC1‐dependent SOCE signaling by elevating TRPC1 mRNA and protein levels. This elevation leads to an increase in the steady‐state intraneuronal calcium concentration ([Ca^2+^] I), heightened ER stress, an imbalance in protein kinase and phosphatase activity, and enhanced tau protein phosphorylation at AT8 and p‐S396, as previously documented.^22^ ER stress leads to the activation of AKT/GSK3β signaling, resulting in robust tau phosphorylation.^113^ Overexpression of hTau decreases the levels of PP2A‐C (the catalytic subunit of PP2A), while PP2A‐B (the regulatory subunit) remains unchanged, as demonstrated in previous research,^22^ PP2A is known to be the primary tau phosphatase, regulating tau phosphorylation at various pathological sites,^114^ This observation suggests that hTau overexpression diminishes PP2A activity, thereby promoting the accumulation of phosphorylated tau (p‐Tau) in the brain. The overexpression of TRPC1 leads to an elevation in phosphorylated CaMKII level, a major kinase that targets the phosphorylation of tau at Ser262, which can be activated by increased intracellular [Ca^2+^].^115^ In summary, overexpression of intracellular hTau can induce ER stress through a TRPC1‐dependent SOCE mechanism, resulting in the dysregulation of protein kinases and phosphatases, ultimately exacerbating tau pathology. Concurrently, TRPC1 overexpression leads to a significant reduction in dendritic spines within the hippocampal CA3 region, resulting in synaptic dysfunction and cognitive deficits.^22^ SOCE‐mediated NFAT1‐NOX2‐NLRP1 inflammasome corpuscle in LPS‐induced neuronal damage and Aβ generation.^116^ C/EBPβ, a transcription factor associated with aging and listed in the GenAge database, is abundantly expressed in the brain and can be activated by risk factors associated with AD.^24^ C/EBPβ was verified as a transcription factor responsible for mediating TRPC1 gene expression (Figure 4). Overexpression of hTau upregulated C/EBPβ expression, subsequently enhancing TRPC1 mRNA transcription and protein expression. Conversely, the downregulation of C/EBPβ reduced hTau‐induced TRPC1 transcription and translation.^22^ The overexpression of hTau leads to an elevation in TRPC1 transcription by activating the transcription factor C/EBPβ, which in turn this upregulation of hTau occurs via the C/EBPβ‐TRPC1‐SOCE signaling pathway, thereby forming another vicious cycle of AD development.

### 2.5 Activation of C/EBPβ induces transcription of the ANP32A gene, suppresses histone acetylation, and stimulates the formation of phosphorylated Tau

**FIGURE 5:**
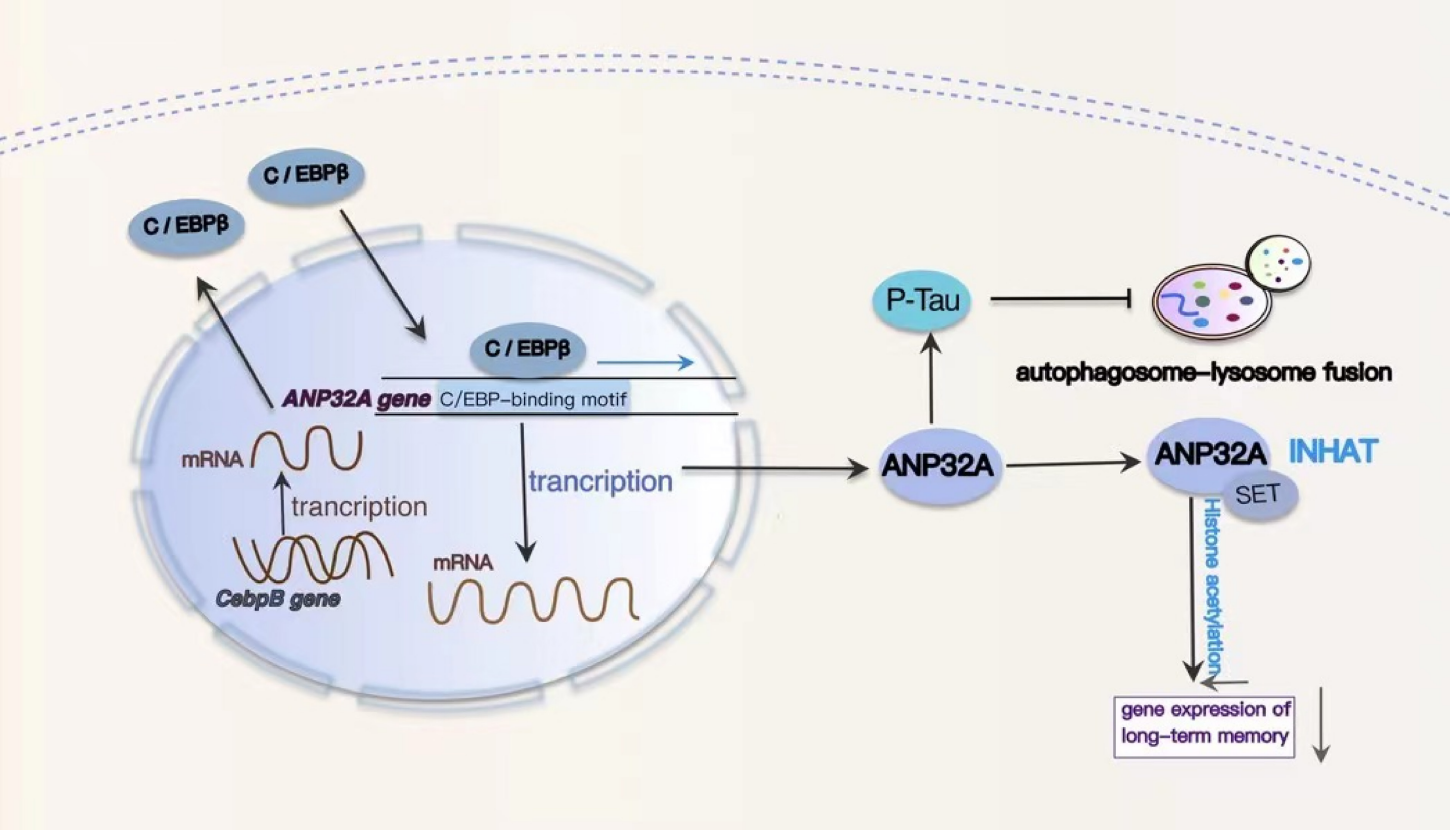
The molecular mechanism of C/EBPβ‐ANP32A‐INHAT/PP2A affecting cognitive function. AD‐related stressors increase total C/EBPβ levels and phosphorylated C/EBPβ levels. C/EBPβ induces ANP32 overexpression by binding to conserved recognition elements in the ANP32A proximal promoter region. ANP32A binds to SET to form an inhibitory complex (INHAT) that binds to histones and blocks their acetylation, ultimately resulting in reduced expression of life‐related genes. ANP32A, as an endogenous PP2A inhibitor, can increase the tau hyperphosphorylation level. AD‐like MAPT/Tau accumulation can inhibit autophagosome‐lysosome fusion by regulating the ANP32A‐INHAT‐IST1‐ESCRT‐III pathway, and overexpressed Tau can further promote *Cebpb* gene expression.

Synaptic damage and memory impairment are hallmark pathologies and symptoms associated with Alzheimer's disease. Epigenetic modifications, particularly protein acetylation within the nervous system, play a crucial role in the regulation of gene expression related to long‐term memory.^117^ Acetylation of histones diminishes the electrostatic attraction between adjacent histones and DNA, thus fostering a more accessible chromatin configuration conducive to the transcription of genes associated with memory.^118^ Histone acetylation, observed to be reduced in animal models of neurodegenerative diseases, including AD, is causally related to cognitive decline.^119^ Histone acetylation and deacetylation processes are catalyzed by histone acetyltransferases (HATS) and histone deacetylases (HDACs), respectively. Additionally, cellular complex acetyltransferase inhibitor (INHAT) inhibits histone acetylation.^120^, ^121^ Two key components of INHAT, ANP32A (also known as I1 PP2A) and SET (also known as I2 PP2A), are selectively upregulated in brain regions affected by neurofibrillary pathology in AD.^122^, ^123^, ^124^, ^125^ ANP32A and SET together form an inhibitory complex, INHAT, which binds to histones and hinders their interaction with HAT.^126^, ^127^ The prevention of ANP32A elevation in the hippocampal CA3 region rescues learning and memory decline in htau transgenic mice, and downregulating ANP32A effectively ameliorates synaptic impairment in these mice.^24^ Meanwhile, the phosphorylation and binding capacity of the microtubule‐associated protein tau are regulated by specific kinases and phosphatases.^128^ The activation of tau kinases, particularly GSK3β, plays a pivotal role in tau hyperphosphorylation in AD and related tauopathies.^129^ PP2A, the primary phosphatase controlling essential neuronal signaling pathways, mainly relies on the B55α subunit and is considered the principal tau phosphatase, accounting for 70% of total tau phosphatase activity.^130^, ^131^, ^132^ The ANP32A and SET act as endogenous PP2A inhibitors, leading to elevated tau hyperphosphorylation levels.^123^, ^124^ Furthermore, the accumulation of AD‐like mitogen‐activated protein kinase (MAPT)/Tau disrupts autophagosome‐lysosome fusion by perturbing the ANP32A‐INHAT‐IST1‐ESCRT‐III pathway (Figure 5). This finding highlights the role of ANP32A in mediating a detrimental cycle of MAPT/Tau accumulation and autophagy deficiency during the chronic progression of AD neurodegeneration.^120^

To further elucidate the underlying mechanisms contributing to the increased expression of ANP32A in AD, we discovered that *ANP32A* mRNA and protein levels were significantly elevated in response to hTau accumulation, Aβ1‐42 exposure, or H_2_O_2_ treatments.^24^ To identify potential regulatory elements, we conducted a screening of *ANP32A* promoter binding sites using the transcription factor binding database,^133^ and we identified a conserved C/EBPβ‐binding site in the proximal promoter region of ANP32A.^24^ Notably, AD‐related stressors, such as tau overexpression, Aβ exposure, and oxidative stress, were found to enhance *ANP32A* gene transcription, with its expression under the regulation of C/EBPβ^24^ (Figure 5). Stimulation of C/EBPβ led to the overexpression of ANP32A.^134^ Additionally, AD‐related stressors were observed to elevate the levels of total and phosphorylated C/EBPβ, which were also increased in the brains of AD patients and transgenic animal models of AD.^31^, ^78^ The upregulation of ANP32A in AD may be a consequence of increased transcription factors C/EBPβ, suggesting that the upregulation of C/EBPβ may represent a key role leading to ANP32A overexpression, followed by histone acetylation and impaired cognition.

### 2.6 C/EBPβ selectively triggers inhibitory GABAergic neuronal degeneration by repressing FOXO1

FOXO, a member of the forkhead family of transcription factors that acts downstream of insulin/insulin‐like growth factor (IGF) signaling, plays conserved roles in longevity, cellular homeostasis, and cognitive performance.^135^ It integrates signals from nutrient deprivation and stress stimuli to coordinate gene programs involved in cellular metabolism and protection against oxidative stress, thereby maintaining organelle and protein homeostasis.^136^, ^137^, ^138^ FOXO is phosphorylated by Akt and Mst1 at residues T24 and S212, respectively. FOXO1 phosphorylated by Akt is sequestered in the cytoplasm, where it inhibits apoptotic genes, such as *BIM* (a mediator of Bcl‐2 cell death), promoting cell survival.^139^ In contrast, MST1‐phosphorylated FOXO1 translocates to the nucleus, where it promotes apoptosis.^134^ REST (RE1 silencing transcription factor, a transcription factor for FOXOs) and nerve activity are associated with IGF signaling, regulating the activity of the tripartite transcription factor. Nuclear REST and FOXO1 levels are strongly positively correlated with genes that repress excitation and synaptic functions, thereby delaying aging and extending lifespan.^140^ The activity of FOXO/DAF‐16 (abnormal DAuer formation‐16) is required to reduce the toxicity of Aβ aggregates under conditions of low insulin signaling,^141^ indicating a potential protective role in AD. C/EBPβ has also been implicated in mediating IGF‐1 and human insulin receptor (IR) expression by binding to its promoter.^16^

**FIGURE 6:**
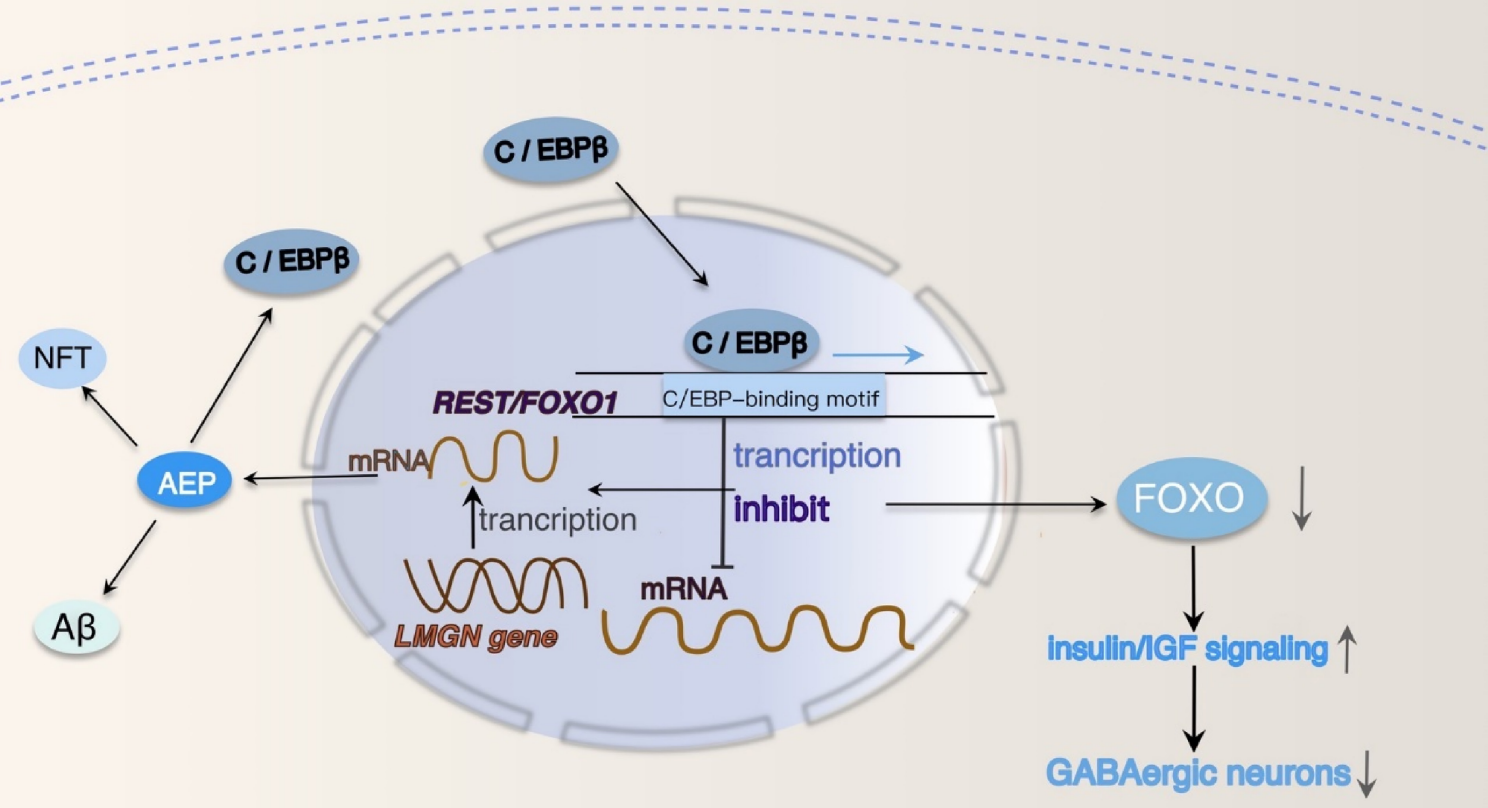
Molecular mechanisms of FOXO interact with C/EBPβ to accelerate AD progression. Overexpression of C/EBPβ inhibits REST and FOXO1 promoter activity and reduces REST and FOXO1 transcription. Decreased FOXO/DAF‐16 (abnormal DAuer formation‐16) activity increased insulin signaling, increased Aβ aggregation toxicity, and induced GABAergic neuronal degeneration and death. Inhibition of *FOXO* gene transcription can promote the transcription of *LGMN*, which leads to the increase of AEP and the accumulation of Aβ and Tau.

The up‐regulation of neuronal C/EBPβ significantly affects insulin‐triggered P‐IR and p‐FOXO1 T24/FOXO1 activity, especially in GABAergic neurons, which renders GABAergic neurons more vulnerable to damage and variability during aging.^23^ The loss of GABA and GABAergic interneurons in AD patients is a significant component of AD and may contribute to the network hyperactivity manifested as seizures.^142^ The onset of seizures frequently precedes cognitive decline and may serve as a precursor to AD.^143^ C/EBPβ and FOXO1 mutually repress each other. C/EBPβ overexpression inhibits *FOXO1*, *FOXO3*, and *REST* mRNA levels, leading to an increase in *Bim* and *LGMN* mRNA levels. Conversely, overexpression of FOXO1 reduces *C/EBPβ* mRNA levels, resulting in a decrease in both *Bim* and *LGMN* mRNAs (Figure 6). Additionally, it has been found that C/EBPβ directly binds to the *REST* and *FOXO1* promoters, functioning as a transcriptional repressor.^23^ C/EBPβ overexpression inhibited the transcription of *FOXO* and *REST* genes by binding to their promoters, which not only makes GABAergic neurons more susceptible to degeneration during aging but also selectively induces the increased expression of *LGMN* in GABAergic neurons and increases the production of AEP in GABAergic neurons, which together lead to the aggravation of cognitive dysfunction in AD.

## 3 MECHANISMS OF ACTION OF C/EBPβ IN OTHER NEURODEGENERATIVE DISEASES

C/EBPβ functions as a transcription factor, contributing to the progression of AD through diverse pathways. There is considerable experimental evidence that C/EBPβ can also contribute to the development of a variety of other degenerative diseases through several pathways. Parkinson's disease (PD) is characterized by the loss of dopaminergic neurons and the presence of intraneuronal Lewy body (LB) inclusions, of which aggregated α‐synuclein (α‐syn) is a major component. C/EBPβ exhibits age‐dependent regulation of MAOB (monoamine oxidase B) expression, a pivotal enzyme in dopamine metabolism, which can trigger oxidative stress in dopaminergic neurons and contribute to the aggregation of α‐syn, a hallmark protein associated with neurodegenerative disorders.^144^ Additionally, C/EBPβ/AEP exhibits age‐dependent activation in Parkinson's disease, wherein it assumes a role in mediating the presence of α‐synuclein in both the gastrointestinal tract and the brain.^145^ Deprivation of C/EBPβ provided substantial in vitro and in vivo neuroprotection to dopaminergic cells, concurrently reducing inflammatory responses and glial activation levels.^146^ The inactivation of the mitochondrial electron transport chain (ETC) activates the C/EBPβ/AEP pathway, thereby triggering the pathogenesis of PD. Deficiencies in all three complexes of the mitochondrial ETC robustly hinder oxidative phosphorylation, leading to a significant increase in ROS levels and activation of the C/EBPβ/AEP pathway, resulting in dopaminergic neuron cell death. C/EBPβ can also affect mitochondrial autophagy by regulating mitochondrial transcription factor A (TFAM).^147^ These deficiencies also contribute to constipation and dyskinesia, key factors in PD pathogenesis.^148^ BDNF and Netrin‐1, which promote neuronal survival and regulate intestinal function, are strongly reduced in both the brain and gut of PD patients, and the deficiency of these nutritional factors in the gut causes dopaminergic neuron loss, constipation, and motor dysfunction. The transcription factor C/EBPβ, induced by inflammation and oxidative stress, acts as a potent repressor of BDNF and Netrin‐1, suppressing the expression of nutritional factors, and thereby triggering non‐motor and motor symptoms of PD. Its level exhibits a negative correlation with BDNF and Netrin‐1 in PD patients.^149^ C/EBPβ preferentially mediates APOE4 expression, which would exacerbate α‐syn pathology.^150^ In multiple sclerosis (MS), the expression level of C/EBPβ is increased, with LAP being the predominant isoform. Loss of C/EBPβ attenuates microglial cell viability and has a neuroprotective effect.^53^ Interaction between myelin basic protein‐primed T cells and microglia activates NF‐κB and C/EBPβ in microglia, leading to the specific expression of proinflammatory cytokines IL‐1β, IL‐1α, TNF‐α, and IL‐1 in microglia.^151^ In amyotrophic lateral sclerosis (ALS), C/EBPβ has also been shown to act as a transcription factor regulating the expression of proinflammatory genes and is a candidate for regulating the expression of potentially neurotoxic genes in ALS microglia.^152^

## 4 C/EBPβ REPRESENTS A PROMISING POTENTIAL THERAPEUTIC TARGET FOR AD TREATMENT

C/EBPβ plays a pivotal role in the modulation of AD‐related protein expression via multiple pathways, making it a crucial target for AD therapy. At present, drugs directly targeting C/EBPβ are relatively scarce in neurodegenerative disease research. However, microglia‐targeting C/EBPβ inhibition has been shown to be a safe and effective method to reduce neurodegeneration driven by neuroinflammation.^53^ There are indications that drug‐induced reduction of C/EBPβ levels may have neuroprotective effects. AD has a higher incidence in elderly women.^153^ Elevated FSH levels following the perimenopausal period directly impact hippocampal and cortical neurons, triggering the expression of *Cebpb*, *Lgmn*, and App, accelerating the deposition of β‐amyloid and Tau, and impairing cognitive function. Blocking FSH eliminated FSH‐induced AD pathology by suppressing the neuronal C/EBPβ‐δ‐secretase pathway, potentially preventing and delaying the progression of AD pathology.^154^ C/EBPβ in microglia is regulated post‐translationally by the ubiquitin ligase COP1 (also called RFWD2). Absent COP1, C/EBPβ rapidly accumulates and instigates a robust pro‐inflammatory and neurodegeneration‐associated gene program. Conversely, COP1 restoration inhibits microglial activation and alleviates neuroinflammation.^19^, ^155^ Although targeting C/EBPβ directly may pose challenges, chemical inducers of degradation could offer a potential approach for modulating C/EBPβ. In a germinal matrix hemorrhage model, Secukinumab mitigated reactive astrogliosis and diminished neurological deficits, in part, through the regulation of the IL‐17RA/C/EBPβ pathways.^156^ Pruni Cortex, a medicinal herb, can suppress the iNOS gene by inhibiting PI3K/Akt signaling and mitigating C/EBPβ phosphorylation, attributed to its constituent, Sakura.^157^ This has noteworthy therapeutic implications for neuroinflammation. Patchouli alcohol (PA) has also been demonstrated to significantly downregulate the protein expression of C/EBPβ and AEP in the hippocampus of Tg mice. This downregulation inhibits the deposition of Aβ plaques, excessive phosphorylation of tau proteins, neuroinflammation, and intestinal ecological imbalance, thereby improving cognitive deficits in the AD mouse model. These findings underscore the promise of PA as a naturally occurring compound deserving of further exploration for its potential in AD pharmacotherapy.^158^ Indeed, in cancer treatment research, numerous natural and synthetic drugs have demonstrated the capacity to inhibit C/EBPβ production, diminish its activity, or facilitate its degradation through targeted approaches. Considering the overexpression of C/EBPβ in AD, it is plausible to anticipate that investigations into drug interventions targeting C/EBPβ hold significant potential for treating AD and other neurodegenerative conditions.

## 5 CONCLUSION

C/EBPβ, activated by inflammatory cytokines, is a pivotal transcription factor regulating key enzymes and proteins across pathways that drive AD progression. Neuroinflammation is a prevalent hallmark of neurodegenerative diseases. C/EBPβ regulates proinflammatory gene expression during glial activation and plays a pivotal role in microglial activation, inducing neurotoxicity.^54^ The δ‐secretase, activated in the aging brain, is the key enzyme in the cleavage of APP and Tau, ultimately leading to cognitive impairment.^63^ C/EBPβ primarily upregulates *LMGN* gene transcription by binding to the CCCAGCTCTGGC promoter sequence, promoting δ‐secretase expression.^79^ *APOE* represents the most potent genetic risk factor for AD. C/EBPβ binds to the *APOE* promoter region (−305 to +93) and primarily mediates the expression of ApoE4 protein induced by Aβ.^20^ C/EBPβ‐mediated vicious cycles occur in all three aforementioned pathways, reinforcing the irreversibility of AD progression. Furthermore, C/EBPβ functions as a transcription factor for the calcium channel protein TRPC1, enhancing C/EBPβ‐TRPC1‐SOCE signaling, leading to ER stress and dysregulation of proteases and phosphatases, resulting in increased phosphorylated hTau and exacerbating AD's pathological progression.^22^ ANP32A, a critical component of INHAT, mediates the vicious cycle between Tau/MAPT accumulation and autophagy deficiency, with its expression primarily regulated by C/EBPβ at the transcriptional level.^24^ Additionally, FOXO, a member of the FOXO transcription factor family, functions downstream of IGF‐like signaling and can be selectively inhibited by C/EBPβ in degenerating GABA neurons.^23^ C/EBPβ promotes Aβ production and increases Tau phosphorylation through multiple pathways, resulting in elevated extracellular Aβ plaques and intraneuronal NFTs, thus mediating a vicious cycle of chronic neuroinflammation and neuronal loss.

The overexpression of C/EBPβ expedites the irreversible advancement of AD pathophysiology. Furthermore, C/EBPβ plays a pivotal role in mediating various pathways associated with AD pathology, some of which develop vicious loops, resulting in the formation of feedback mechanisms. The significant involvement of C/EBPβ in prevalent degenerative diseases such as PD, MS, and ALS has been established. Additionally, drugs that counteract C/EBPβ in cancer therapy have shown promising experimental outcomes.^159^ For instance, Sesquiterpene lactone is frequently employed in cancer treatment research as a natural compound. Notably, Helenalin acetate can bind to the initial 21 amino acids of LAP*, disrupting its interaction with the coactivator p300 and consequently suppressing the transcription of the target gene.^160^ Withaferin A and Celastrol have likewise been demonstrated to inhibit C/EBPβ activity through a mechanism similar to that of Helenalin acetate.^161^ ST101, an innovative and selective peptide antagonist of C/EBPβ, binds to the leucine zipper domain of C/EBPβ, preventing its dimerization and augmenting the ubiquitin‐proteasome‐dependent degradation of C/EBPβ.^162^ Research has demonstrated that LIP promotes cell survival during staurosporine‐ or taxol‐induced Hep3B cell death.^163^ Targeting LAP*/LAP could potentially result in LIP overexpression. Consequently, this review aims to enhance our comprehension of the interplay between C/EBPβ signaling and the expression of pertinent target genes. It also underscores the potential of C/EBPβ as a therapeutic target for addressing AD and other degenerative conditions. Currently, our understanding of the regulatory mechanisms governing gene expression in pathogenic glial cells remains limited, and the effectiveness and potential side effects of C/EBPβ antagonists in neurodegenerative diseases remain unclear. Researchers can continue to explore C/EBPβ as a focal point for extensive and in‐depth investigations, with the aim of developing more efficacious treatment strategies and pharmaceutical interventions for AD, other neurodegenerative disorders, and even more challenging diseases.

## AUTHOR CONTRIBUTIONS

QY and CBL drafted the manuscript, PCY, GYZ, WW, XQR, and JY contributed to the analysis of the results, and FZH and WDL reviewed and modified the manuscript. All authors read and approved the final manuscript.

## FUNDING INFORMATION

This work was supported by grants from the National Natural Science Foundation of China (82271234; 82060219); Natural Science Foundation of Jiangxi Province (20212ACB216009; 20212BAB216048); Jiangxi Province “Double Thousand Plan” (jxsq2019201023); Youth Team Project of the Second Affiliated Hospital of Nanchang University (2019YNTD12003).

## CONFLICT OF INTEREST STATEMENT

The authors declare no conflict of interest.

## CONSENT FOR PUBLICATION

All authors read and approved the final manuscript.

## Data Availability

Not applicable.
